# Chloroplast Genome Sequence of *Lagerstroemia guilinensis* (Lythraceae, Myrtales), a Species Endemic to the Guilin Limestone Area in Guangxi Province, China

**DOI:** 10.1128/genomeA.00341-16

**Published:** 2016-05-19

**Authors:** Cuihua Gu, Luke R. Tembrock, Zhiqiang Wu

**Affiliations:** a School of Landscape and Architecture, Zhejiang Agriculture and Forestry University, Lin’an, Hangzhou, Zhejiang, People’s Republic of China; b Department of Biology, Colorado State University, Fort Collins, Colorado, USA

## Abstract

We announce here the first complete chloroplast genome sequence of *Lagerstroemia guilinensis* (Lythraceae, Myrtales), a species endemic to the Guilin limestone area, along with its genome structure and functional gene annotations. The plant was collected from Guilin, Guangxi, China, and deposited as a germplasm accession of the Zhejiang Agriculture and Forestry University Collection (ZAFU 1507144). This genome will provide valuable information for future research of the *Lagerstroemia* genus and its relatives.

## GENOME ANNOUNCEMENT

*Lagerstroemia* is the most economically valuable genus in *Lythraceae* due to its utility as an ornamental plant. The genus is composed of about 55 species (1–3). *Lagerstroemia guilinensis* is a 2-m-tall shrub with a distribution documented only around Xishan Park in Guilin City, China. Due to its narrow and limited distribution, *L. guilinensis* is at a higher risk of extinction than other broadly distributed *Lagerstroemia* species. *L. guilinensis* is only found growing on limestone mountains and blooms from May until July. Molecular research has been done to identify *Lagerstroemia* cultivars and interspecific hybrids (4, 5), but there is a lack of complete genome-level research on *Lagerstroemia*. We acquired *L. guilinensis* (ZAFU 1507144) samples from Xishan Park of Guilin City, Guangxi Province, China, to finish its chloroplast (cp) genome.

Chloroplast genomes have a highly conserved circular DNA quadruplet structure ranging from 120 to 165 kb, with conserved gene order, similarity of sequence across the land plants, uniparental inheritance, and low recombination rates (6–8) compared to nuclear genomes. Plant cp genomes provide a valuable resource of markers in phylogenetics (9–11), DNA barcoding (12), and biogeography among populations (13). With the dramatically reduced cost of next-generation sequencing, it has become more convenient to sequence whole cp genomes (14). More than 900 land plant complete cp genomes can be accessed at the NCBI database (15).

The raw Illumina reads generated for this report were trimmed by quality score using Trimmomatic version 0.3 (16). The *de novo* assembly of reads from *L. guilinensis* were finished using CLC Genomics Workbench version 7, with the default settings (CLC bio). *De novo* assembly was used (17) to construct the assemblies. After merging Illumina and Sanger sequence data, the whole cp genome for *L. guilinensis* was found to be 152,074 bp. The final cp genome was annotated by DOGMA (http://dogma.ccbb.utexas.edu/) with manual adjustment of the exon-intron junctions (17).

We elucidated the genomic characteristics of this species: the cp genome was 152,074 bp in length, with 37.6% overall G+C content. The genome structure was highly similar to that of land plants, consisting of two inverted regions (IRs) (25,677 bp), a large single copy (LSC) (83,811 bp), and a small single copy (SSC) (16,909 bp). Of the 112 unique genes (78 protein-coding genes, 4 rRNAs, and 30 tRNAs), 82 genes are located in the LSC region (60 protein-coding genes and 22 tRNA genes), 13 genes are located in the SSC region (12 protein-coding genes and 1 tRNA gene), and 17 genes are located in both IR regions (6 coding genes, 4 rRNA genes, and 7 tRNA genes). Sixteen genes were found to have introns, with 5 tRNA genes having a single intron each (*trnA-GUC, trnG-UCC, trnI-GAU, trnK-UUU, trnL-UAA*, and *trnV-UAC*), eight protein-coding genes having a single intron each (*atpF, ndhA, ndhB, petB, petD, rpl16, rpoC1*, and *rps16*), and three protein-coding genes having two introns each (*clpP*, *rps12*, and *ycf3*).

### Nucleotide sequence accession number. 

The accession number for the DNA sample of *L. guilinensis* was stored at Zhejiang Agriculture and Forestry University. The complete cp genome sequence with its annotation has been submitted to GenBank under the accession no. KU885923.

## References

[1] Qin HN, Graham SA 2007 Lagerstroemia. Flora of China 13:277–281. http://www.efloras.org/florataxon.aspx?flora_id=2&taxon_id=117489.

[2] Koehne E 1884 Lythraceae monographice describuntur. Morphologie de Vegetationsorgane. Botanical Jahrbücher für Systematik, Pflanzengeschichte und Pflanzengeographie 4: Beiblatt 2, Heft 1:95–132.

[3] Furtado CX, Srisuko M 1969 A revision of *Lagerstroemia* L. (*Lythraceae*). Gdns Bull 24:185–335.

[4] Pooler MR 2003 Molecular genetic diversity among 12 clones of *Lagerstroemia fauriei* revealed by AFLP and RAPD markers. HortScience 38:256–259.

[5] Pounders C, Rinehart T, Sakhanokho H 2007 Evaluation of interspecific hybrids between *Lagerstroemia indica* and *L*. *speciosa*. HortScience 42:1317–1322.

[6] Palmer JD 1985 Comparative organization of chloroplast genomes. Annu Rev Genet 19:325–354. doi:10.1146/annurev.ge.19.120185.001545. 3936406

[7] Ravi V, Khurana JP, Tyagi AK, Khurana P 2008 An update on chloroplast genomes. Plant Syst Evol 271:101–122. doi:10.1007/s00606-007-0608-0.

[8] Wicke S, Schneeweiss GM, DePamphilis CW, Müller KF, Quandt D 2011 The evolution of the plastid chromosome in land plants: gene content, gene order, gene function. Plant Mol Biol 76:273–297. doi:10.1007/s11103-011-9762-4. 21424877 PMC3104136

[9] Moore MJ, Bell CD, Soltis PS, Soltis DE 2007 Using plastid genome-scale data to resolve enigmatic relationships among basal angiosperms. Proc Natl Acad Sci USA 104:19363–19368. http://dx.doi.org/10.1073/pnas.0708072104. doi:10.1073/pnas.0708072104. 18048334 PMC2148295

[10] Wang L, Qi XP, Xiang QP, Heinrichs J, Schneider H, Zhang XC 2010 Phylogeny of the paleotropical fern genus *Lepisorus* (Polypodiaceae, Polypodiopsida) inferred from four chloroplast DNA regions. Mol Phylogenet Evol 54:211–225. doi:10.1016/j.ympev.2009.08.032. 19737617

[11] Wu ZQ, Ge S 2012 The phylogeny of the BEP clade in grasses revisited: evidence from the whole-genome sequences of chloroplasts. Mol Phylogenet Evol 62:573–578. doi:10.1016/j.ympev.2011.10.019. 22093967

[12] Day A, Goldschmidt-Clermont M 2011 The chloroplast transformation toolbox: selectable markers and marker removal. Plant Biotechnol J 9:540–553. doi:10.1111/j.1467-7652.2011.00604.x. 21426476

[13] Wang W, Messing J 2011 High-throughput sequencing of three *Lemnoideae* (duckweeds) chloroplast genomes from total DNA. PLoS One 6:e24670. doi:10.1371/journal.pone.0024670. 21931804 PMC3170387

[14] Soltis DE, Gitzendanner MA, Stull G, Chester M, Chanderbali A, Chamala S, Jordon-Thaden I, Soltis PS, Schnable PS, Barbazuk WB 2013 The potential of genomics in plant systematics. Taxon 62:886–898.

[15] Wu ZQ, Tembrock LR 2015 Two complete chloroplast genomes of white campion (*Silene latifolia*) from male and female individuals. Mitochondrial DNA 1736:1–2. doi:10.3109/19401736.2015.1126829. 26714215

[16] Bolger AM, Lohse M, Usadel B 2014 Trimmomatic: a flexible trimmer for Illumina sequence data. Bioinformatics 30:2114–2120. doi:10.1093/bioinformatics/btu170. 24695404 PMC4103590

[17] Wu ZQ, Tembrock LR, Ge S 2015 Are differences in genomic data sets due to true biological variants or errors in genome assembly: an example from two chloroplast genomes. PLoS One 10:e0118019. doi:10.1371/journal.pone.0118019. 25658309 PMC4320078

